# The content of Recovery College courses in England: a 71 college document analysis

**DOI:** 10.3389/fpsyt.2025.1605498

**Published:** 2025-06-17

**Authors:** Simran Kaur Takhi, Holly Hunter Brown, Amy Ronaldson, Vanessa Lawrence, Merly McPhilbin, Benjamin Rose Ingall, Riddhi Daryanani, Jonathan Simpson, Tesnime Jebara, Simon Lawrence, Agnieszka Kapka, Yasuhiro Kotera, Danielle Dunnett, Daniel Hayes, Katy Stepanian, Caroline Fox Yeo, Sara Meddings, Jane Rennison, Katherine Barrett, Jason Grant Rowles, Yuki Miyamoto, Hans Kroon, Mariam Namasaba, Claire Henderson, Mike Slade

**Affiliations:** ^1^ School of Health Sciences, Institute of Mental Health, University of Nottingham, Nottingham, United Kingdom; ^2^ Health Service and Population Research Department, Institute of Psychiatry, Psychology, and Neuroscience, King’s College London, London, United Kingdom; ^3^ Center for Infectious Disease Education and Research, Osaka University, Osaka, Japan; ^4^ Research Department of Behavioural Science and Health, Institute of Epidemiology and Health Care, University College London, London, United Kingdom; ^5^ Buildings, Energy & Environment Research Group, Department of Architecture & Built Environment, University of Nottingham, Nottingham, United Kingdom; ^6^ Imroc Head Office, Nottingham, United Kingdom; ^7^ RECOLLECT Lived Experience Advisory Panel (LEAP), King’s College London, London, United Kingdom; ^8^ Department of Psychiatric Nursing, Graduate School of Medicine, The University of Tokyo, Bunkyo-Ku, Tokyo, Japan; ^9^ Tranzo Scientific Center for Care and Well-being, School of Social and Behavioural Sciences, Tilburg University, Tilburg, Netherlands; ^10^ Trimbos Institute, Netherlands Institute of Mental Health and Addiction, Utrecht, Netherlands; ^11^ Faculty of Nursing and Health Sciences, Nord University, Bodø, Norway

**Keywords:** Recovery college, document analysis, fidelity measure, course content, inductive content analysis

## Abstract

**Introduction:**

Recovery Colleges (RCs) exist in 28 countries and across five continents. The concept of recovery and recovery-oriented care has become widespread internationally and embedded in policy documentation and mental health services. As a result, Recovery Colleges, which focus on adult learning and co-production, have now developed a global presence, but many psychiatrists are unfamiliar with this intervention. RCs can be categorized as ‘Strengths Oriented’, focusing on skills and knowledge development, or ‘Community-oriented’, emphasizing strengthening community and social connections. Research has not sufficiently investigated RC curriculum and how course provision differs depending on RC orientation. The study aimed to develop a typology of RC courses and assess differences in course types across RC orientations.

**Method:**

A document analysis was conducted. The websites of 88 RCs in England were searched to collect online prospectuses. Overall, 2,330 courses described in 551 documents from 71 RCs were collated. Inductive content analysis was applied to the course titles to develop a typology of courses offered. Mann-Whitney U tests were used to assess differences in the median number of course types offered by Strengths-Oriented versus Community-Oriented colleges.

**Results:**

A typology of 14 superordinate course categories was created. The three most common course categories were Self-management of Well-being (96% RCs ≥1 course, median 10 courses per RC), Mental Health Conditions and Symptoms (85% RCs ≥1 course, 4 courses per RC), and Creativity (86% RCs ≥1 course, 3 courses per RC). The least common course categories included Issues relating to the Extended Support Network and Issues relating to Staff (38% RCs ≥1 course, 0 courses per RC) (6% RCs ≥1 course, 0 courses per RC). The median number of courses did not differ between Strengths-oriented versus Community-oriented RCs, with the exception of more Practical Life Skills (p=0.021) and Involvement, Co-production and Research (p=0.036) courses in Strengths-oriented RCs.

**Conclusions:**

RCs support mental health recovery through a diverse curriculum. Community-facing and strengths-based, health service-affiliated RCs offer similar courses. RCs prioritize equipping students with knowledge about living with mental health issues. Courses targeted to informal carers are lacking. Further cross-cultural extension of the typology is needed.

## 1 Introduction

Recovery colleges (RCs) support people experiencing mental health issues through an adult learning model which is co-produced by people with lived experience of mental health issues and those with mental health expertise (1). Individuals with lived experience of mental health issues and mental health expertise, as well as informal carers and staff, can enroll onto RC courses as students. RC courses are open to all, including people with lived experience of mental health challenges, those with professional expertise, and carers, each participating equally as students in a shared learning environment. RCs are shaped by the principles of mental health recovery, moving away from prioritizing symptom reduction and towards supporting the development of the skills and resources needed to live a meaningful life alongside mental health issues (2). Beyond individual benefits, RCs also have a broader impact on mental health organizations and systems. RCs have been found to promote attitudinal shifts among mental health professionals (e.g. increased recognition of the strengths of those with lived experience of mental health issues), reduce stigma through wider public engagement with mental health education and encourage more recovery-oriented practices among staff and services (3).

Whilst several countries endorse recovery oriented practice e.g. Ireland’s National Framework for Recovery in Mental Health (4), Quebec’s Mental Health Action Plan (5), and Australia’s National Framework for Recovery Oriented practice (6), RCs are only recently mentioned as a specific innovation (7) and are absent from national mental health policy including in England (8). Despite this lack of central planning, RCs are becoming globally available. A global survey in 2022 identified 221 RCs operating in 28 countries (9) with the highest numbers in England.

RCs have defining principles: co-production and adult learning (10). Co-production refers to people with lived experience being active agents in contributing to all elements of the RC including operation, curriculum development and quality assurance (11). RC courses are thus co-designed, co-produced and co-facilitated between people with professional and lived experience of mental health issues. Adult learning refers to self-directed learning, where people engage in courses for their self-development, and learn from each other as well as from the staff who deliver the courses (12).

RCs are used by diverse mental health service user populations. A single site evaluation of an RC in England which compared the student cohort to those using the local mental health services and the local population found that students were more representative of the population than the trust caseload was in terms of ethnicity and sexual orientation (13). The benefits of RCs within wider society have been evidenced. Psychiatrists have identified that RCs positively shift the power dynamics between professionals and service users by emphasizing collaboration and reciprocal relationships across the RC and wider lived experience community (14). RCs can be transformational in the way mental health professionals understand their service users. Mental health professionals who participated in RC courses have explained having an enhanced comprehension of the needs and challenges of their service users as a result of course attendance (15).

Two important knowledge gaps exist regarding RC courses. First, the range of course content is unknown. Courses vary widely across RCs, due to the emphasis on local course development intended to meet student needs. Course content typically encompasses skills and knowledge acquisition in relation to understanding mental health issues and treatment, self-management of difficulties, development of social networks and living healthier and more independent lives outside of services. Examples of course include *Top Tips for Applications and Interviewers, Understanding Autism, and Sleep Hygiene.*

Each college develops its own curriculum, and each course is co-produced within individual RCs. Co-production of courses at an individual RC level means that RCs are constantly contributing to a culture of parity between qualified mental health professionals and service users through power sharing – a key RC principle. No frameworks exist to standardize content, either for topics to cover or for content of individual courses. RC staff have discussed the sharing of course materials in networks such as the International Recovery College Learning Set, a platform that facilitates knowledge sharing and continual development among RCs.

Whilst flexibility of course content is a valued aspect of RCs, this comes at a price. A nationwide survey conducted in England (16) found that the mean cost of creating a course was UK£8,101 and of running an existing course was UK£2,111. In other words, the cost of co-producing one new local course is the same cost as running an existing course four times. Informed decision-making about the optimal balance between new and existing courses whilst maintaining co-production of courses requires an understanding of the types of courses run in RCs.

The second knowledge gap is the relationship between course content and RC characteristics. Key RC characteristics have been established in the Recovery Colleges Characterisation and Testing (RECOLLECT) study (17). The RECOLLECT Fidelity Measure is a 12-item manager-rated quantitative assessment measuring the extent to which key recovery-oriented principles are present in a specific RC (10). The measure assesses seven modifiable and five non-modifiable characteristics. Seven non-modifiable components correspond to values-based organizational components, including Valuing Equality, Learning Tailored to the Student, Co-production, Social Connectedness, Community Focus and Commitment to Recovery. A further five modifiable components evaluate RC characteristics which vary across colleges: Available to all, Location, Distinctiveness of Course Content, Strengths-based and Progressive. A cluster analysis based on RECOLLECT Fidelity Measure data and other RC characteristics (such as location, length of operation and average number of students) was conducted to identify how RCs could be distinguished from one another (16). RCs differed significantly on two organizational characteristics (location, main organizational affiliation) and six student characteristics including sex and whether students were using mental health services. RCs were found to be clustered into three distinct groupings: Strengths-oriented colleges (affiliated with the mental health service, and explicitly focus on amplifying strengths of students), Community-oriented colleges (use community rather than statutory health service buildings and place a strong focus on foster social connectedness) and Forensic colleges (cater to forensic, mainly male, populations, and have an implicit focus on amplifying strengths).

Research has not identified a typology of courses offered in RCs. A typology refers to the classification of observations based on qualitative or quantitative analysis (18) in order to classify phenomenon into discrete yet interrelated categories (19). The absence of a course typology hinders the ability to investigate how the provision of courses link with college characteristics and student outcomes. The aims of this study were to develop the first typology for courses offered by RCs in England, and to investigate differences in course provision between Strengths-oriented and Community-oriented RCs.

## 2 Materials and methods

### 2.1 Design

A document analysis of publicly available documents describing courses from all RCs in England, using the four-step READ methodology: I, ready your materials, II extract data, III analyze data and IV, distil your findings (20).

### 2.2 Procedures

The sample of RCs was derived from a 2021 national survey of all RCs in England (16). The inclusion criteria for the survey comprised any organization in England that focuses on supporting personal recovery and aspires to use co-production and adult learning approaches. RCs were identified using web searches, expert consultation with RC national leaders, existing RC networks, snowball sampling, and liaison with host charities and health services. A total of 88 RCs in England were identified, of which 63 (72%) participated in the national survey, meaning that their RC orientation (Strengths-oriented, Community-oriented or Forensic) could be identified.

### 2.3 Data collection and analysis

The READ method of document analysis was followed (20). READ Step I involved identifying the nature and number of documents to be analyzed based on the research objectives. To develop a comprehensive typology, all 88 RCs identified within the national survey were included, irrespective of whether they participated in the survey. Inclusion criteria comprised publicly available information on RC websites describing any educational offer, such as courses, workshops, and pre-booked or drop-in sessions (collectively termed ‘courses’). Documents describing either individual courses or whole prospectuses were obtained from the public websites of each RC and were collated by HHB and MM in July 2022. Documents describing courses offered before 2022 were excluded, as were courses in Forensic RCs due to the lack of online documents.

READ Step II involved extraction and collation. Each course title was extracted, along with the name of the RC and the name of the document describing the course so the course description could be located. In READ Step III, inductive content analysis, a process utilizing inductive and deductive approaches was used to develop the initial typology (21). Two researchers (HHB and SKT) developed the first version of the typology by independently reviewing 400 course titles and noting a list of courses that were appearing across the titles (e.g. “outdoor leisure activities”). Microsoft Word was used to collate emerging categories of courses. Whilst software tools can be used to conduct content analysis, these were not utilized in the study as the approach emphasizes that the thought process of inductive content analysis (ICA) is the same, regardless of the tool (21). The researchers used a combination of Microsoft word and oral discussions (via Microsoft Teams) to facilitate the analysis. Regular meetings were held to compare and discuss categories, resolve discrepancies, and begin to group categories with common attributes into broader higher order categories. Once the typology was refined, deductive analysis was conducted to evaluate how well the typology encompassed course content. In this pilot phase, seven analysts independently applied the typology to categorize the same one hundred course titles. Each analyst read a course title and assigned a category from the typology. Where the course title was ambiguous, such as ‘Human Needs,” the document describing the course content was read before assigning a course category. An adequate rater concordance of 0.84 was found. Following the pilot round of using the typology deductively on the one hundred course titles, discussions were held between analysts to highlight discrepancies in coding, refine the framework and maximize rater concordance. Once the typology was finalized (**Supplementary Material 1**), all of the remaining 2330 courses titles were deductively coded by the 7 analysts.

In READ Step IV4, findings were summarized to investigate the relationship between RC orientation (Strengths-oriented versus Community-oriented as identified in our previous national survey of RCs) (16) and courses. Each course title was tabulated with RC name, orientation (and typology Category). The number of times each superordinate theme was seen across course offerings from each of the 71 RCs, was noted on an Microsoft Excel sheet.17 RCs were not involved in the national survey (16) and were excluded from statistical analysis. These 17 RCs were solely used to develop the typology.

The median number of courses per college was calculated, comprising the middle number in a sorted list of course numbers across all RCs. The dispersion of course numbers was assessed using interquartile range (IQR), in this case indicating the range of the middle half of course frequency, i.e. the second and third quartiles. Higher IQR indicates low consistency in the number of courses offered.

RCs with different orientations (Strengths-oriented versus Community-oriented) were assessed using Mann-Whitney U tests. Forensic orientation RCs and RCs with no orientation data were not included in this part of the analysis. The statistical significance threshold was set at 0.05, and Bonferroni adjustment for multiple testing was used.

## 3 Results

A total of 2,330 courses were identified across 551 documents, coming from 71 (81%) of the 88 known RCs in England. Reasons for not obtaining documents from the remaining 17 RCs available included no documents available (n=8), no eligible documents available (n=8), and RC being shutdown (n=1).

### 3.1 Typology of RC courses

A summary of the course content typology with definitions of the 14 superordinate categories is shown in **Table 1**, along with the median number of courses per RC coded to that category and the proportion of RCs offering at least one course coded to that category. The full coding framework comprising 14 superordinate categories and 53 subordinate categories is shown in **Supplementary 1**.

**Table 1:** Summary typology of courses (n=2,330) provided by 71 recovery colleges in England.

Superordinate category and definition	Subordinate category examples	Course title examples	Median courses per college (Median IQR)	Proportion of RCs (N=71) offering the course n (%)
**1. Well-being Self-management** Learning how to manage well-being	Self-care skills. Fostering Self Compassion.	Improving Self-esteem and Confidence. How Can We Make Self-care Happen? Life After Stroke.	10 (15-16)	68 (96)
**2. Mental Health conditions and symptoms** Learning about mental health conditions/symptoms and how to live with/manage them.	Anxiety Disorders and or Symptoms. Self-harm and Suicide	Understanding Phobias, Coping With Anxiety. Understanding Hoarding.	4 (1 to 8)	60 (85)
**3. Creativity** Learning about or taking part in creative writing, arts and crafts, musical activities, and performance.	Creative Writing. Literature, and Story Telling.	Journaling for Well-being. Poetry. Tree of life.	3 (1 to 6)	61 (86)
**4. Physical Health** Learning about the role of physical health or an opportunity to engage in physical health activities.	Sleep Hygiene. Exercise. Stretching and Yoga.	Dance for Fun. Happiness, and Health. Exploring Sleep.	2 (1 to 4)	58 (82)
**5. Social Connection** Learning about social skills relating to relationships and communication, opportunities to engage in social activities.	Recreation, Team Games and Opportunities for connection.	Loneliness: get better connected. Communication 101. Let’s chat coffee morning.	1 (0 to 3)	51 (72)
**6: Practical Life skills** Developing skills and knowledge for practical aspects of life.	Money management and finances. Housing.	Nutrition and Budgeting. Applications and interviews. Getting comfortable with Zoom.	1 (0 to 3)	48 (66)
**7. Nature and Outdoors** Opportunities to engage in outdoors activities.	Opportunities to Learn about or engage in gardening. Courses involving animals.	Guided Walking Trail Through <city>. Green Prescription: Growing Plants for Well-being. Mindfulness in Nature.	1 (0 to 2)	36 (51)
**8. Identity** Learning about identities such as sexuality or gender.	Learning About Identity-specific Issues.	Understanding the LGBTQ+ Community. Gender and Me. Gender, sexuality and mental health.	0 (0 to 2)	32 (45)
**9. Treatments and Interventions** Learning about medication or types of therapy.	Medication. Therapy.	Coming off Medication and Discontinuation Effects. Dialectical Behaviour Therapy (DBT) Skills Refresher. An Introduction to Compassion Focused Therapy.	0 (0 to 1)	27 (38)
**10. Involvement, Co-production and Research** Learning about, or opportunities for involvement in co-production activities and sharing lived experience.	Involvement, Co-Production and Research.	Using your Lived Experience and Getting Involved. Peer Tainer Training. What is co-production?	0 (0 to1)	26 (37)
**11. Qualifications** Accredited and non-accredited qualifications	Qualifications	Understanding Quality Improvement – Bronze Training. Mental health First Aid. Level 2 Counselling skills in Loss.	0 (0 to 1)	22 (31)
**12. Stigma** Learning about different types of stigma, dealing with conscious/unconscious biases.	Stigma and Prejudice.	Understanding Unconscious Bias. Stamping Out Mental Health Stigma Dispelling myths: Bipolar Disorder.	0 (0 to 1)	14 (20)
**13. Issues Related to the Extended Support Network** Courses for friends, family and loved ones.	Courses for Loved Ones of Those Mental Health Issues.	Health and Well-being for Carers, Family and Friends. Caring & mental Health: Mental Health Support for Carers.	0 (0 to 1)	27 (38)
**14. Issues Related to Staff**	Courses relating to staff	Staff Well-being. What is WRAP? Information for Staff and Supporters. Finding Peace in a Busy Day (wellbeing retreats for healthcare staff).	0 (0 to 0)	4 (6)

**Figure 1:**
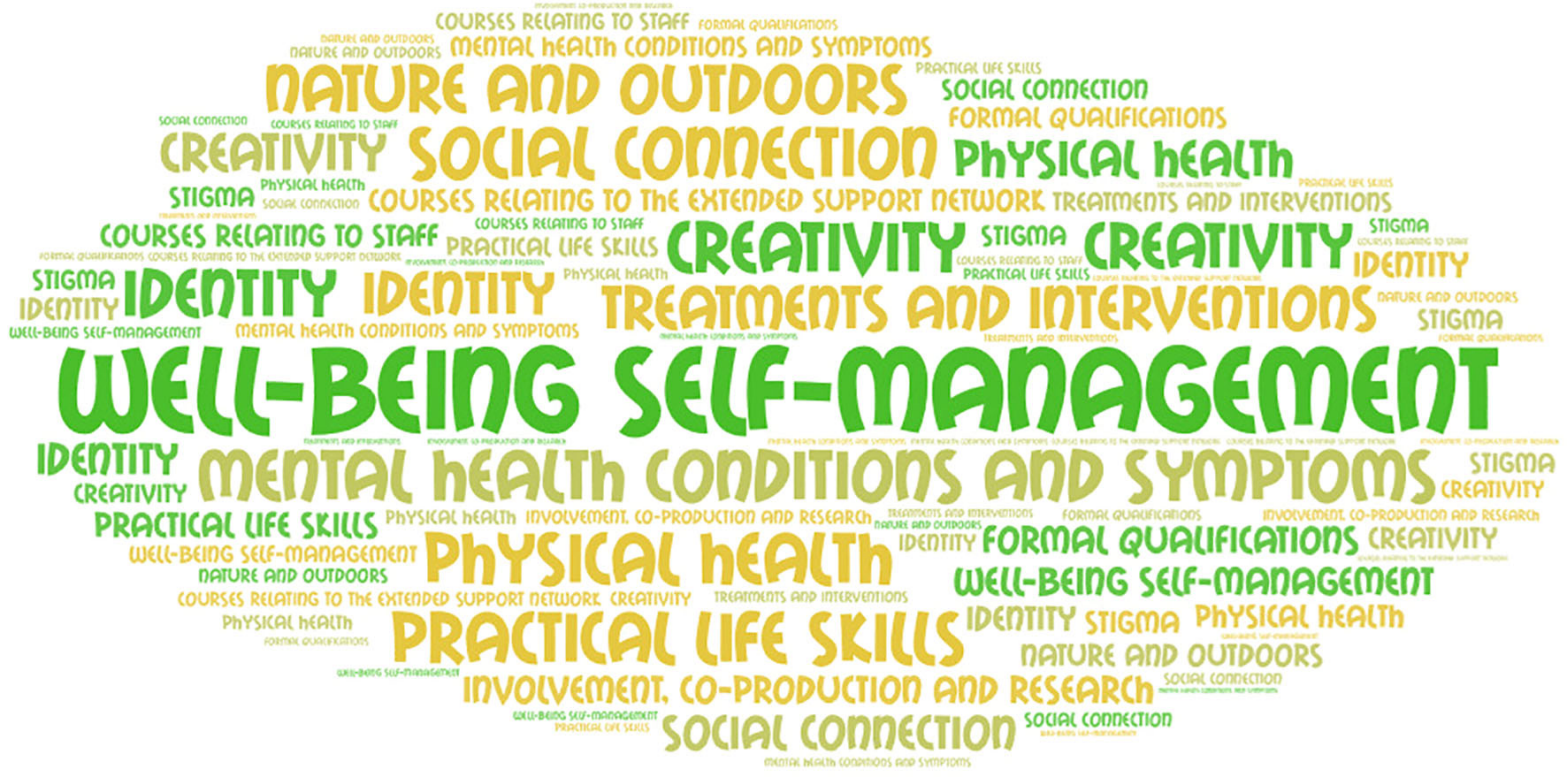
Superordinate themes by frequency.

The most common categories were Self-management of Well-being (offered in 96% of RCs, median 10 courses per RC), Mental Health Conditions and Symptoms (85% of RCs, median 4 per RC), and Creativity (86% of RCs, median 3 per RC). Other courses offered in more than 50% of RCs were Physical health (82%), Social Connection (72%), Practical Life Skills (66%), and Nature and Outdoors (51%). The frequency of each course category is shown diagrammatically in **Figure 1**.

### 3.2 Differences in course content across RC orientations

The orientation of 54 (76%) of the 71 included RCs was available from national survey data and comprised 39 Strengths-oriented and 15 Community-oriented colleges. This allowed 1,922 (82%) of 2,330 courses to be associated with an RC orientation, comprising 1,466 courses in Strengths-oriented RCs and 456 in Community-oriented RCs. The median number of most types of course were similar across Strengths-oriented and Community-oriented, with the exceptions of Practical life skills (p=0.021) and Involvement (p=0.036), co-production and research which were both higher in Strengths-oriented colleges. After adjustment, neither difference was significant. The comparision of courses in strengths-oriented vs community oriented Recovery Colleges is shown in **Table 2**.

**Table 2:** Comparison of courses in strengths-oriented (n=39) versus community-oriented (n=15) recovery colleges.

Course	Proportion of RCs offering the course n (%)		Median number of courses per RC median (IQR)		
Orientation	Strengths	Community	Strengths	Community	*p*
n	*39*	*15*	*1,466*	*456*	≤ 0.05
1. Well-being self-management	39 (100)	15 (100)	11 (6 to 17)	10 (3 to 12)	0.223
2. Mental health conditions and symptoms	35 (90)	14 (94)	4 (3 to 8)	4 (1 to 8)	0.265
3. Creativity	33 (85)	14 (94)	4 (1 to 6)	4 (2 to 7)	0.593
4. Physical health	33 (85)	15 (100)	2 (2 to 4)	3 (1 to 4)	0.714
5. Social connection	29 (74)	9 (60)	2 (0 to 3)	1 (0 to 4)	0.744
6. Practical life skills	33 (85)	6 (40)	2 (1 to 4)	0 (0 to 2)	0.021*
7. Nature and outdoors	19 (49)	8 (53)	0 (0 to 2)	1 (0 to 2)	0.708
8: Practical life skills	33 (85)	6 (40)	2 (1 to 4)	0 (0 to 2)	0.021*
9. Treatments and interventions	19 (49)	4 (27)	0 (0 to 1)	0 (0 to 1)	0.200
10. Involvement, co-production and research	20 (51)	2 (13)	1 (0 to 2)	0 (0 to 0)	0.036*
11. Qualifications	11 (28)	4 (27)	0 (0 to 1)	0 (0 to 1)	0.813
12. Stigma	10 (26)	2 (13)	0 (0 to 1)	0 (0 to 0)	0.381
13. Courses Related to the Wider Support Network	16 (41)	5 (33)	0 (0 to 1)	0 (0 to 1)	0.383
14. Courses Related to Staff	4(10)	0 (0)	0 (0 to 0)	0 (0 to 0)	0.520

## 4 Discussion

The three most common course categories were Self-Management of Well-being, Mental Health Conditions and Symptoms, and Creativity, with the three least common course categories being Issues Relating to Staff, Issues relating to Informal Carers and Qualifications. Overall, the typology indicates that RCs offer a diverse curriculum facilitating new knowledge and self-management skills, which are both directly targeting and indirectly supportive of improved mental health. The courses span all of the Connectedness-Hope-Identity-Meaning-Empowerment (CHIME) Framework of recovery processes (22). Most courses being categorized under Self-Management of Well-being is consistent with research illustrating English RCs as emphasizing self-management courses within promotional texts (23). Courses provided at Strength-based RCs which are affiliated with the mental health system are similar in profile to courses at community-oriented RCs, with a greater emphasis on practical living skills and involvement activities in the former. The course typology demonstrates that RC courses across both strengths-oriented and community-oriented RCs provide a range of recovery support. Courses such as Improving Self-esteem and Confidence and Understanding Phobias may target cognitive and affective change, whereas courses such as *Life After Strok*e, *Nutrition and Budgeting*, *and Green Prescription: Growing Plants for Well-being* could target more behavioral and functional change. RCs have evidenced cognitive changes where students no longer identify themselves as merely unwell individuals and instead, have transitioned to viewing themselves as responsible for their own recovery (24). Some courses focus on social inclusion outcomes, either directly through courses such *as Exploring Job Searching Techniques*, *Getting Comfortable with Zoom*, and *How to Say No*, or indirectly by encouraging students to connect with each other, e.g*., Guided Walking Trail Through* <city>. Others focus on rights-based knowledge, such as *Mental Health Disclosure*: *Know Your Rights* and *Understanding Unconscious Bias* 1, or on experiential learning such as *Learning to Play Guitar*. Many courses coded under Creativity contained therapeutic components. For example, *Journalling for Well-being* courses involves students reflecting on their existing resources, aspirations and life goals.

The most widely offered course types are consistent with the RC focus on recovery and well-being, with all RCs running courses on supporting well-being. Whilst the process of evaluation of RCs is evolving and few evaluation studies evidence those with lived experience of mental health issues co-producing such research (25), Well-being is frequently used as the outcome domain by which RC effectiveness is evaluated (26), for example, both in-person (27) and online (28).

RCs are empowering environments which use co-produced adult learning approaches to support self-management and community integration. The RECOLLECT Multi-Level Change model (29) identifies the key RC change mechanisms as Empowering Environment, Shifting the Balance of Power, Facilitating Personal Growth, and Enabling Different Relationships. Furthermore, a study of RC students identified five key mechanisms of change a judgment-free environment; supportive relationships, mutuality and role modelling; deconstruction of self-stigma; and reclaiming of one’s power. (30). RCs support recovery-oriented care (31) which is associated with less stigmatizing attitudes toward mental health issues (3). Stigma-awareness within RCs is shown by the empowering language used in course titles and by course content addressing prejudices, e.g., Dispelling Myths about Bipolar.

Some types of course are infrequently offered. The paucity of accredited, formal educational offers suggests that the principle that RCs should ‘act as a conduit towards mainstream learning and training opportunities in the community rather than a segregated alternative’ (p.6) (32) is shared across the RC community. Courses explicitly designed for members of a person’s support network (e.g. spouses, parents, children) are less commonly offered. The lack of carer-focused courses may reflect the complexities of shaping the RC offer around the needs of carers e.g. a preference for courses outside of working hours (33). Despite few courses relating to the wider support network, many courses are inclusive of all roles, reflecting the RC principle of shared learning among peers, carers, and professionals.

Other less-frequently offered courses support personal transformation, e.g. involvement in co-production, identity change, addressing stigma, and life skills to manage life demands. These capture a range of approaches to engaging specific mechanisms of action relating to personal growth and enabling different relationships.

This study has three research and practice implications. First, the typology enables courses to be compared across RCs. Observing similar courses across different RCs would highlight course variation to inform decision-making around the value-for-money offered by locally developing each course versus developing a pool of key high pedagogical-quality courses for use across multiple RCs. A balance needs to be struck between tailoring course content to specific populations (34) and maximizing efficiency of time and resources through information sharing between RCs. For example, where most RCs are offering a similar type of course, course templates could be developed as a shared resource, whilst still encouraging RCs to co-produce their courses. Our typology can be used to inform reflection on how best RCs can avoid wasteful duplication whilst maintaining an ethos of co-production.

Second, RCs have a shared focus on recovery and well-being but vary in the courses they offer to promote mechanisms of action. Evaluation and comparison of the impact of different types of courses targeting the same mechanism, e.g. facilitating personal growth or enabling different relationships, will be needed to inform future development of courses across the RC community. Future research could explore how specific course details link with RC and student outcomes. Although RCs have been linked to mechanisms and outcomes such as empowerment, and building supportive relationships, no research to date has directly investigated how specific types of courses drive particular outcomes. Establishing these direct links between course content, mechanisms of change, and student outcomes represents an important and promising direction for future investigation. The typology provides a defensible classification allowing mapping of course type to specific mechanism(s) of action.

Finally, the fidelity-level distinction between Strengths-oriented and Community-oriented RCs does not appear to impact on course content. This may reflect shared recovery principles or shared structural constraints within the English RC commissioning and operating environment. This may mean that an understanding of the implications of RC orientation for courses needs to be more detailed and focused on how the course is delivered, for example through in-course observation. The current evidence indicates that future investigation of courses may not need to balance the participating sample by RC orientation.

### 4.1 Strengths and limitations

The strengths of the study include nationwide coverage, the use of a formal document analysis methodology, and multi-analyst involvement to reduce bias. In terms of study limitations, all course data were extracted in July 2022. This narrow timeframe may have missed documents that were temporarily unavailable due to being updated. A second limitation is the geographical reach of this typology. While the typology is comprehensive, qualitative analysis was based primarily on course titles, with full course descriptions consulted only when the title was ambiguous. As a result, further details such as the number of sessions, structure of the courses, or the proportion of course time dedicated to different topics, could not be systematically analyzed. Although this approach enabled consistent categorization across a large dataset, it limits the ability to draw more granular insights into course delivery and emphasis. The proportion of course time or curriculum listing devoted to each category of course would provide alternative metrics of the presence of each course across the RC community.

All RCs in England were in scope, but RCs now exist in 28 countries (9). Future research could focus on refining the typology through document analysis of RCs in other countries. Culture influences RC operation (35). A 28-country study identified that three cultural characteristics – higher individualism, higher indulgence and lower uncertainty avoidance – were associated with higher fidelity (36). A study of 169 RCs across the world identified seven specific aspects of fidelity which were impacted on by these three cultural characteristics: equality, learning, co-production, community focus, commitment to recovery, strengths-based practice and course distinctiveness (37). An analysis of course documentation in England and Japan found that relational and long-term aspects of recovery in Japan are emphasized, compared with a focus on self-management and skills acquisition in England (23). It would be important to replicate the study in other jurisdictions as this would enable the assessment of the breadth and depth of course offerings, allowing planners and managers to assess factors such as inclusivity. Cross-cultural validation of the typology would also allow investigation of the impact of how differing healthcare structures, organizational characteristics and conceptualizations of recovery, impact on courses. For example, in some regions of the world, there may be a need for courses which integrate indigenous approaches to healing. Just as with psychological therapies, cultural adaption may be needed.

## 5 Conclusion

RC courses primarily focus on well-being, understanding mental health issues and using creative approaches to support recovery. RCs use diverse course types to support recovery, and if cross-culturally-validated, this typology offers a resource for existing and new RCs to consider the diversity of their curriculum, and a theoretical foundation for evaluating course influences on student outcomes.

## Data Availability

The raw data supporting the conclusions of this article will be made available by the authors, without undue reservation.
