# Correction: The content of Recovery College courses in England: a 71 college document analysis

**DOI:** 10.3389/fpsyt.2025.1652479

**Published:** 2025-07-08

**Authors:** Frontiers Production Office

**Affiliations:** Frontiers Media SA, Lausanne, Switzerland

**Keywords:** Recovery college, document analysis, fidelity measure, course content, inductive content analysis

Reference 15 was incorrectly cited in place of reference 20. The correct citation has now been inserted in the section **2 Materials and methods**, *2.3 Data collection and analysis*, Paragraph 1 and should read:

“The READ method of document analysis was followed (20).”

A sentence was repeated. A correction has been made to the section **2 Materials and methods**, *2.3 Data collection and analysis*, Paragraph 2:

“Following the pilot round of using the typology deductively on the one hundred course titles, discussions were held between analysts to highlight discrepancies in coding, refine the framework and maximize rater concordance.”

Reference 24 was cited in the wrong sentence and should have been cited in place of reference 25. A correction has been made to **4 Discussion**, Paragraph 1:

“Courses such as Improving Self-esteem and Confidence and Understanding Phobias may target cognitive and affective change, whereas courses such as *Life After Strok*e, *Nutrition and Budgeting*, *and Green Prescription: Growing Plants for Well-being* could target more behavioral and functional change. RCs have evidenced cognitive changes where students no longer identify themselves as merely unwell individuals and instead, have transitioned to viewing themselves as responsible for their own recovery (24).”

The number of key mechanisms of change was incorrectly given as four. A correction has been made to the section **4 Discussion**, Paragraph 3:

“Furthermore, a study of RC students identified five key mechanisms of change.”

There was a mistake in **Table 1** as published. Some course title examples were omitted. The corrected **Table 1** appears below.

**Table 1:** Summary typology of courses (n=2,330) provided by 71 recovery colleges in England.

Superordinate category and definition	Subordinate category examples	Course title examples	Median courses per college (Median IQR)	Proportion of RCs (N=71) offering the course n (%)
**1. Well-being Self-management** Learning how to manage well-being	Self-care skills. Fostering Self Compassion.	Improving Self-esteem and Confidence. How Can We Make Self-care Happen? Life After Stroke.	10 (15-16)	68 (96)
**2. Mental Health conditions and symptoms** Learning about mental health conditions/symptoms and how to live with/manage them.	Anxiety Disorders and or Symptoms. Self-harm and Suicide	Understanding Phobias, Coping With Anxiety. Understanding Hoarding.	4 (1 to 8)	60 (85)
**3. Creativity** Learning about or taking part in creative writing, arts and crafts, musical activities, and performance.	Creative Writing. Literature, and Story Telling.	Journaling for Well-being. Poetry. Tree of life.	3 (1 to 6)	61 (86)
**4. Physical Health** Learning about the role of physical health or an opportunity to engage in physical health activities.	Sleep Hygiene. Exercise. Stretching and Yoga.	Dance for Fun. Happiness, and Health. Exploring Sleep.	2 (1 to 4)	58 (82)
**5. Social Connection** Learning about social skills relating to relationships and communication, opportunities to engage in social activities.	Recreation, Team Games and Opportunities for connection.	Loneliness: get better connected. Communication 101. Let’s chat coffee morning.	1 (0 to 3)	51 (72)
**6: Practical Life skills** Developing skills and knowledge for practical aspects of life.	Money management and finances. Housing.	Nutrition and Budgeting. Applications and interviews. Getting comfortable with Zoom.	1 (0 to 3)	48 (66)
**7. Nature and Outdoors** Opportunities to engage in outdoors activities.	Opportunities to Learn about or engage in gardening. Courses involving animals.	Guided Walking Trail Through <city>. Green Prescription: Growing Plants for Well-being. Mindfulness in Nature.	1 (0 to 2)	36 (51)
**8. Identity** Learning about identities such as sexuality or gender.	Learning About Identity- specific Issues.	Understanding the LGBTQ+ Community. Gender and Me. Gender, sexuality and mental health.	0 (0 to 2)	32 (45)
**9. Treatments and Interventions** Learning about medication or types of therapy.	Medication. Therapy.	Coming off Medication and Discontinuation Effects. Dialectical Behaviour Therapy (DBT) Skills Refresher. An Introduction to Compassion Focused Therapy.	0 (0 to 1)	27 (38)
**10. Involvement, Co-production and Research** Learning about, or opportunities for involvement in co-production activities and sharing lived experience.	Involvement, Co- Production and Research.	Using your Lived Experience and Getting Involved. Peer Tainer Training. What is co-production?	0 (0 to1)	26 (37)
**11. Qualifications** Accredited and non-accredited qualifications	Qualifications	Understanding Quality Improvement – Bronze Training. Mental health First Aid. Level 2 Counselling skills in Loss.	0 (0 to 1)	22 (31)
**12. Stigma** Learning about different types of stigma, dealing with conscious/unconscious biases.	Stigma and Prejudice.	Understanding Unconscious Bias. Stamping Out Mental Health Stigma. Dispelling myths: Bipolar Disorder.	0 (0 to 1)	14 (20)
**13. Issues Related to the Extended Support Network** Courses for friends, family and loved ones.	Courses for Loved Ones of Those Mental Health Issues.	Health and Well-being for Carers, Family and Friends. Caring & mental Health: Mental Health Support for Carers.	0 (0 to 1)	27 (38)
**14. Issues Related to Staff**	Courses relating to staff	Staff Well-being. What is WRAP? Information for Staff and Supporters. Finding Peace in a Busy Day (wellbeing retreats for healthcare staff).	0 (0 to 0)	4 (6)

There was a formatting error in reference 11. It should be:

“Thornhill H, Dutta A. Are recovery colleges socially acceptable? BJPsych Int. (2016) 13:6–7. doi: 10.1192/s2056474000000878”.

The original version of this article has been updated.

