# Correction: The content of Recovery College courses in England: a 71 college document analysis

**DOI:** 10.3389/fpsyt.2025.1674921

**Published:** 2025-09-05

**Authors:** Simran Kaur Takhi, Holly Hunter Brown, Amy Ronaldson, Vanessa Lawrence, Merly McPhilbin, Benjamin Rose Ingall, Riddhi Daryanani, Jonathan Simpson, Tesnime Jebara, Simon Lawrence, Agnieszka Kapka, Yasuhiro Kotera, Danielle Dunnett, Daniel Hayes, Katy Stepanian, Caroline Fox Yeo, Sara Meddings, Jane Rennison, Katherine Barrett, Jason Grant Rowles, Yuki Miyamoto, Hans Kroon, Mariam Namasaba, Claire Henderson, Mike Slade

**Affiliations:** ^1^ School of Health Sciences, Institute of Mental Health, University of Nottingham, Nottingham, United Kingdom; ^2^ Health Service and Population Research Department, Institute of Psychiatry, Psychology, and Neuroscience, King’s College London, London, United Kingdom; ^3^ Center for Infectious Disease Education and Research, Osaka University, Osaka, Japan; ^4^ Research Department of Behavioural Science and Health, Institute of Epidemiology and Health Care, University College London, London, United Kingdom; ^5^ Buildings, Energy & Environment Research Group, Department of Architecture & Built Environment, University of Nottingham, Nottingham, United Kingdom; ^6^ Imroc Head Office, Nottingham, United Kingdom; ^7^ RECOLLECT Lived Experience Advisory Panel (LEAP), King’s College London, London, United Kingdom; ^8^ Department of Psychiatric Nursing, Graduate School of Medicine, The University of Tokyo, Bunkyo-Ku, Tokyo, Japan; ^9^ Tranzo Scientific Center for Care and Well-being, School of Social and Behavioural Sciences, Tilburg University, Tilburg, Netherlands; ^10^ Trimbos Institute, Netherlands Institute of Mental Health and Addiction, Utrecht, Netherlands; ^11^ Faculty of Nursing and Health Sciences, Nord University, Bodø, Norway

**Keywords:** Recovery college, document analysis, fidelity measure, course content, inductive content analysis

In the published article, there was an error in the author list, and author Mariam Namasaba was erroneously excluded. The corrected author list appears below.

“Simran Kaur Takhi1*, Holly Hunter Brown2, Amy Ronaldson2, Vanessa Lawrence2, Merly McPhilbin2, Benjamin Rose Ingall2, Riddhi Daryanani2, Jonathan Simpson2, Tesnime Jebara2, Simon Lawrence2, Agnieszka Kapka2, Yasuhiro Kotera1,3, Danielle Dunnett2, Daniel Hayes4, Katy Stepanian2, Caroline Fox Yeo5, Sara Meddings6, Jane Rennison6, Katherine Barrett7, Jason Grant Rowles7, Yuki Miyamoto8, Hans Kroon9,10, Mariam Namasaba2, Claire Henderson2† and Mike Slade1,11†”

The article citation has been updated to read:

“Takhi SK, Brown HH, Ronaldson A, Lawrence V, McPhilbin M, Ingall BR, Daryanani R, Simpson J, Jebara T, Lawrence S, Kapka A, Kotera Y, Dunnett D, Hayes D, Stepanian K, Yeo CF, Meddings S, Rennison J, Barrett K, Rowles JG, Miyamoto Y, Kroon H, Namasaba M, Henderson C and Slade M (2025) The content of Recovery College courses in England: a 71 college document analysis. *Front. Psychiatry* 16:1605498. doi: 10.3389/fpsyt.2025.1605498”

The copyright statement has been updated to read:

“© 2025 Takhi, Brown, Ronaldson, Lawrence, McPhilbin, Ingall, Daryanani, Simpson, Jebara, Lawrence, Kapka, Kotera, Dunnett, Hayes, Stepanian, Yeo, Meddings, Rennison, Barrett, Rowles, Miyamoto, Kroon, Namasaba, Henderson and Slade. This is an open-access article distributed under the terms of the Creative Commons Attribution License (CC BY). The use, distribution or reproduction in other forums is permitted, provided the original author(s) and the copyright owner(s) are credited and that the original publication in this journal is cited, in accordance with accepted academic practice. No use, distribution or reproduction is permitted which does not comply with these terms.”

The **Author Contributions** section has been updated to read:

“ST: Writing – review & editing, Writing – original draft, Formal analysis. AR: Formal analysis, Writing – review & editing. VL: Methodology, Writing – review & editing. MM: Data curation, Formal analysis, Writing – review & editing. BI: Writing – review & editing, Formal analysis. RD: Writing – review & editing, Formal analysis. JS: Writing – review & editing, Formal analysis. TJ: Writing – review & editing. SL: Writing – review & editing. AK: Writing – review & editing. YK: Writing – review & editing. DD: Writing – review & editing. DH: Writing – review & editing. KS: Writing – review & editing. CY: Writing – review & editing. SM: Writing – review & editing. JR: Writing – review & editing. KB: Writing – review & editing. JR: Writing – review & editing. RW: Writing – review & editing. YM: Writing – review & editing. HK: Writing – review & editing. MN: Writing – review & editing. CH: Supervision, Writing – review & editing, Conceptualization. MS: Writing – review & editing, Supervision, Conceptualization.”

The original article has been updated.

