# Supplementary material for: The content of Recovery College courses in England: a 71 college document analysis
Source: Front Psychiatry. 2025 Jun 17;16:1605498. doi: 10.3389/fpsyt.2025.1605498 (PMC12209312; doi:10.3389/fpsyt.2025.1605498)
Supplement: Supplementary file 1 [file Table1.docx]

Supplementary Material

**Supplement 1. Full Typology of courses (n=2,330) provided by 71 Recovery Colleges in England**

| **­­Theme** | **Definition** | **Example Course titles** |
| --- | --- | --- |
| **Theme 1: Mental health conditions and symptoms** | **Learning about mental health conditions/symptoms and how to live with or manage them.** |  |
| 1.1 Mood-related Diagnoses or Symptoms | Learning about mood disorders or symptoms, and how to live with or manage them, e.g. low mood, depression, bipolar disorder. | Dispelling Myths: Bipolar Disorder. Coping with Low Mood. Introduction to Managing Depression. |
| 1.2 Personality Disorder Diagnoses or Symptoms | Learning about personality disorders or symptoms, and how to live with or manage them, e.g. emotionally unstable personality disorder/borderline personality disorder, antisocial personality disorder. | Understanding Personality disorders. Understanding Emotionally Unstable and Borderline Personality Disorder. Understanding Personality Disorder and Complex Emotional Needs. |
| 1.3 Anxiety Disorders or Symptoms | Learning about anxiety disorders or symptoms, and how to live with or manage them, e.g. anxiety, phobias, generalized anxiety disorder, agoraphobia, social anxiety, panic attacks. | Reducing Anxiety. Coping with Anxiety and Panic Disorder. Understanding Phobias. |
| 1.4 Eating Disorders or Diagnoses | Learning about eating disorders or symptoms, and how to live with or manage them, e.g. anorexia, bulimia, binge eating. | Dispelling Myths: Anorexia. Raising awareness around Eating Disorders**.** Managing Binge Eating |
| 1.5 Dissociative Diagnoses or Symptoms | Learning about dissociative disorders or symptoms, and how to live with or manage them, e.g. dissociative identity disorder, derealisation. | Dissociation. Delirium Awareness. |
| 1.6 Psychosis diagnosis or symptoms | Learning about psychotic disorders or symptoms, and how to live with or manage them, e.g. hallucinations, psychosis, schizophrenia, hearing voices. | Understanding a diagnosis of psychosis and schizophrenia. Living with psychosis and delusions. Managing psychosis in challenging times. |
| 1.7 Obsessive Compulsive Diagnosis or Symptoms | Learning about obsessive compulsive behaviours and disorders, and how to live with or manage them, e.g. hoarding, OCD, dysmorphic disorders. | Understanding Hoarding. Decluttering for Wellbeing. |
| 1.8 Substance use and addictive disorder diagnoses | Learning about addiction, and how to live with or manage them, e.g. alcohol use, drug use, and smoking without specific reference to addiction. | Understanding and managing addiction and dual diagnosis. Smoking facts and fiction. Gambling: what’s the problem? |
| 1.9 Self-harm and Suicide | Learning about self-harm and suicidality, and how to live with or manage it. | Suicide Awareness (including boundaries).Suicide Prevention Training. Lets Talk about Suicide |
| 1.10 Complex or Long-term Trauma and Adversity | Learning about or discussing trauma, complex or long-term trauma and adversity generally, or specific experiences of trauma and adversity, e.g. domestic abuse, bullying, FGM, PTSD. | Trauma and Mental health. Overcoming the Effects of Bullying. Trauma and the Body. |
| 1.11 Degenerative symptoms | Learning relating to degenerative health conditions, and how to live with or manage them. | Understanding Dementia. “I forgot…” - Exploring Memory. Living with Dementia. Talking about Dementia  Arthritis and you. Fibromyalgia Support Group. |
| 1.12 Physical Health conditions and physical disabilities | Learning about physical health conditions, disabilities and how to live with or manage them. | Life after Stroke. Chronic Fatigue. |
| 1.13 Neurodevelopmental conditions | Learning about neurodevelopmental conditions, e.g. ADHD, Autism. | Understanding Autism. ADHD. The Neurodiversity Workshop. |
| **Theme 2: Treatments and interventions** | **Learning about medication or types of therapy.** |  |
| 2.1 Medication | Learning about psychiatric medication, what it is used for, how it may help, and which side effects it is associated with. | Know your medication. Medication explored. Coming off medication and discontinuation effects. |
| 2.2 Therapy | Learning about different therapies/how to apply them, e.g. counselling, CBT, EMDR. | Dialectical Behaviour Therapy (DBT) Skills Refresher. What's it like - Cognitive Analytic Therapy. What Is CBT? |
| **Theme 3: Creativity** | **Learning about or taking part in creative writing, arts and crafts, musical activities and performance.** |  |
| 3.1 Creative Writing, Literature, and Story telling | Learning about or opportunities to take part in creative writing, journaling, storytelling, or personal narratives. | Poetry. Exploring Literature. Journaling for Wellbeing. |
| 3.2 Arts and Crafts | Learning about or opportunities to create visual art or engage with crafts, e.g. painting, embroidery, woodworking. | Painting. Colouring for Wellbeing. Photography. |
| 3.3 Performing arts | Learning about or opportunities to take part in performing arts. | Drama for confidence. Lights drama action. A play made by you. |
| 3.4 Music | Learning about or opportunities to take part in musical activities. | Learning to play the guitar. Music for wellbeing. Music appreciation. |
| **Theme 4: Physical health** | **Learning about the role of physical health or an opportunity to engage in physical health activities.** |  |
| 4.1 Stretching and yoga | Learning about the role of physical health or an  opportunity to engage in physical health activities. | Chair Yoga. Kundalini Yoga. Stretch to Start the Week on Zoom. |
| 4.2 Exercise | Learning about the role of, or an opportunity to engage in, physical exercise or sports.  *[Does not include walking; coded in 6.3]* | Couch to 5K. Dance for Fun. Happiness, and Health. Tai Chi |
| 4.3 Sleep Hygiene | Courses related to learning about the importance of sleep, improving sleep, and sleep hygiene skills. | Exploring Sleep. The Value of Sleep. Dreaming of a Better sleep. |
| 4.4 Diet, Nutrition, and Cooking | Courses relating to eating well and improving cooking skills. | Nutrition and Budgeting. Mood, Food and Mental Health. How Healthy Rating and Being Active can Improve Mental Health. |
| **Theme 5: Social Connection** | **Learning about social skills relating to relationships and communication, opportunities to engage in social activities.** |  |
| 5.1 Social relationships skills | Learning about social skills relating to relationships and communicating. | Everyday Leadership. Exploring Validation. How to Say No |
| 5.2 Recreation, Team Games and Opportunities for Connection and Communication | Courses providing opportunities to socially connect. Also includes open discussion of any topics. | Coffee Morning. Perspectives: Focused Topic Discussion workshop. Table Tennis. Fun with Numbers. |
| **Theme 6: Nature and outdoors** | **Opportunities to engage in outdoors activities.** |  |
| 6.1 Gardening | Opportunities to Learn bout or engage in gardening. | The Allotment Project. Live to grow – grow to live. Green prescription: growing plants for wellbeing. |
| 6.2 Animals | Courses involving animals, opportunities to spend time with animals, or how animals can improve wellbeing. | Horse wisdom for well-being. Our animal teachers. Animal Communications -  an intuitive communication |
| 6.3 Walking | Courses involving walking/activities with a walking component. | Mindful walking and journalling. Photography walk. Making strides. |
| 6.4 Day trips and cultural enrichment | Day trip involving being outdoors with nature or a cultural activity, e.g. going to a museum or historical site | Tour of <city> Castle. Walking the seasons (Botanic Gardens <city>) Guided walk a trail through <city>’s industrial coal mining past. |
| **Theme 7: Wellbeing self-management** | **Learning how to self-manage difficulties.** |  |
| 7.1 General wellbeing | Learning about management of personal wellbeing. This theme also encompasses courses tackling multiple aspects of wellbeing, e.g. mind, body and spirit in one session. | Foundations For Good Mental Health. Mind Body and Spirit. Making the Most of Each Day. Fundamentals of Wellness. Human Needs. |
| 7.2 Self-care Skills | Learning about self-care and tips on how to manage individual self-care. | Self-Care: Why It Is Important and How Do We Make it Happen? Self Care and Management. Enhancing Self Care and Wellbeing |
| 7.3 Thoughts and Emotions | Learning about emotions/ learning therapeutic skills for managing and regulating emotions and thoughts. | Emotional Skills Course. Understanding Anger. Positive Thinking for Stress Reduction. |
| 7.4 Dealing with Personal Difficulties | Learning about challenges, difficulties and stressors that life can bring up, which may not be related to mental health conditions. | Living with Grief and Loss. Dealing With Stress. Reframing Loneliness. |
| 7.5 Fostering Self-compassion | Learning about how to improve self-compassion. | Discovering Self-compassion. Being Kind to Yourself. Compassionate Voice. |
| 7.6 Self-esteem and Self-confidence | Learning about the role of self-esteem and -confidence, and how to improve these. | Building Confidence. Body confidence. Knowing our worth. |
| 7.7 Spirituality, meditation and mindfulness | Opportunities to learn about the role of spirituality, mindfulness and meditation in wellbeing, and opportunities to practice these skills. | Practical Mindfulness. Spirituality and Recovery. Introduction to Spirituality and Wellness. |
| 7.8 Resilience and Self-Discovery | Learning about resilience and/or how to improve personal resilience. Also encompasses content relating to understanding/developing strengths and understanding yourself. *[Note that the title may not reference the word “resilience” specifically.]* | Intro to building resilience. Tree of life. Bouncebackability. Understanding yourself. Myers Briggs. |
| 7.9 Recovery Journey | Learning about/facilitating recovery and the mental health journey. | Recovery: the new me. Meet the inner critic. Health and wellbeing. |
| 7.10 Goal setting | Learning about goal setting and opportunities to set goals. | Introduction to Goal Setting. Setting SMART goals.    Goal Setting for Recovery |
| **Theme 8: Practical Life skills** | **Developing skills and knowledge for practical aspects of life.** |  |
| 8.1 Money management and Finances | Learning about money management, how to save money and or opportunities to create personal budgets. | Life on a Budget. Money matters: making the most of your income. Dealing with debt. |
| 8.2 Housing | Learning about housing issues, from understanding housing rights and benefits to the impact of housing on wellbeing and recovery. | Housing benefits. Better health, better housing. Managing housing needs. |
| 8.3 Employment and volunteering | Learning about employment and volunteering, learning about skills needed for employment or volunteering, support with finding a role, or opportunities to volunteer. | Job Applications. Top tips for Applications and Interviews. Getting the Most from Volunteering. |
| 8.4 IT Support and Internet Safety | Opportunities for IT support or learning about IT topics, including using software and staying safe on social media. | Getting Comfortable with Zoom. IT support. Social Media Safety. |
| 8.5 Understanding Your Rights and How to Advocate for Yourself | Learning about rights, entitlements and how to advocate for yourself around various issues. | Know Your Renter’s Rights Workshop. Mental health Act and Personal Rights. Culturally Appropriate self-Advocacy. |
| **Theme 9: Stigma** | **Learning about different types of stigma, dealing with conscious/unconscious biases.** |  |
| 9.1 Stigma and Prejudice | Courses which involve learning about different types of stigma, conscious/unconscious biases and how to deal with it. | Understanding Stigma. Understanding Unconscious Bias. Stamping Out Mental Health Stigma. |
| **Theme 10: Identity** | **Learning about identities such as sexuality or gender.** |  |
| 10.1 Learning about identities | Learning about identities such as sexuality or gender. | Understanding LGBTQ. Lesbian or Gays in need of Support. Being Me! LGBTQ+ & Allies |
| 10.2 Learning About Identity-specific Issues | Learning about or support for issues which impact a specific group of people. | Let’s discuss…menopause. Culture masculinity. Men’s health & wellbeing. |
| 10.3 Courses for Specific Groups of People | Courses targeted to a particular demographic e.g. gender, elderly. *[Do not code for courses aimed at carers/family]* | All4Men. Domestic Abuse & Mental Health (Women only). Andy Mans Talking group. |
| **Theme 11: Involvement, Co-production and Research** | **Learning about, or opportunities for involvement in, co-production activities and sharing lived experience.** |  |
| 11.1 Involvement, co-production and research | Learning about, or opportunities for involvement in co-production activities and sharing lived experience between those with mental health issues and illness.  Also includes educating others on research happening in the trust. | Involvement workshop. Student Forum Meeting. Your Lived Experience and Getting involved. |
| **Theme 12: Education**  **and Qualifications** | **Accredited and non-accredited educational courses.** |  |
| 12.1 Education and qualifications | Educational courses and qualifications that may or may not be accredited and non-accredited. | Positive Psychology. Introduction to Philosophy. Understanding Quality Improvement – Bronze training. |
| **Theme 13: Courses for Wider Support Network** | **Courses for friends, family and loved ones.** |  |
| 13.1 Courses for informal carers | Courses specifically for those who care/know loved one with mental health issues and illness. | Looking after yourself - health and wellbeing for carers, family and friends. Caring & mental health: mental  health support for carers. |
| 13.2 Courses relating to supporting/ being a carer to someone with lived experience of mental illness and issues | Courses about supporting loved ones with mental health conditions. | Workshops for Family and Friends Supporting a Loved One Living with Mental Health Difficulties. |
| **Theme 14: Courses for staff** | **Courses for health service staff.** |  |
| 14.1 Courses for Staff | Courses for mental health staff. | Staff Wellbeing. Nurturing Self-compassion. What is WRAP information for Staff and Supporters. |
